# Multimetallic Mesoporous Spheres Through Surfactant‐Directed Synthesis

**DOI:** 10.1002/advs.201500112

**Published:** 2015-06-25

**Authors:** Bo Jiang, Cuiling Li, Masataka Imura, Jing Tang, Yusuke Yamauchi

**Affiliations:** ^1^ World Premier International (WPI) Research Center for Materials Nanoarchitectonics (MANA)National Institute for Materials Science (NIMS)1–1 NamikiTsukubaIbaraki305‐0044Japan; ^2^ Faculty of Science and EngineeringWaseda University3–4–1 OkuboShinjukuTokyo169‐8555Japan

**Keywords:** mesoporous materials, mesoporous metals, multimetallic, self‐assembly, surfactants

## Abstract

**Multimetallic mesoporous spheres** are successfully synthesized with ultra‐large mesopores with the assistance of nonionic triblock copolymer (F127) as a structural directing agent. The kinetically controlled reduction rate of metal species and the concentration of F127 are critical to the formation of the large mesopores.

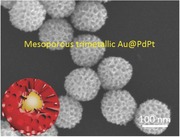

Noble metal nanostructures have received much attention due to their broad applications in hydrogenation reactions, coupling reactions, fuel cells, and localized surface plasma resonances, etc.^1^ Thanks to previously extensive efforts, noble metal catalysts have made significant progress in the applications of fuel cells for solving the problem of energy crisis. To enhance catalytic performance in fuel cells, most of the previous studies not only focused on the composition but also concentrated on the nanostructure of catalysts. In particular, Pt‐based multi­metallic architectures such as dendritic core–shell Au@Pt nano­particle,^2^ polyhedral PtCu_3_ alloy,^3^ hollow Pt–Pd nanocage,^4^ hollow Pt–Ni nanosphere,^5^ PtPdTe nanowires,^6^ and Pt‐on‐Pd_0.85_Bi_0.15_ nanowires^7^ have represented as emerging classes of nanomaterials, showing excellent performances compared with irregular Pt nanoparticles as well as monometallic Pt.

Among various Pt‐based metallic nanostructures, the preparation of mesoporous Pt‐based nanostructure is considered as one of the effective strategies to improve the electrochemical activity owing to their high surface areas, abundant active sites, and accessible pores. In this study, our target is to synthesize Pt‐based “multimetallic” mesoporous spheres to enhance the electrochemical activity toward methanol oxidation reaction (MOR) and reduce the usage of Pt at the same time. Soft‐ and hard‐templating methods have been proposed so far to synthesize mesoporous metals.^8^ Solution phase synthesis is also recognized as an environmentally friendly approach and easily extended to large‐scale production.^9^ However, it should be noted that most of previously reported mesoporous metal materials are based on monometallic Pt nanostructures.^10^ In most cases, the mesoporous metals possess randomly arranged and small‐sized pores, which seriously inhibits effective circulation of reactant/product species within the pores.

Therefore, to explore a facile and reliable strategy for preparation of multimetallic mesoporous spheres with ultra‐large mesopores is of great importance for both fundamental research and practical applications. Here, we have successfully synthesized bimetallic PdPt spheres with ultra‐large mesopores by surfactant‐directing method. Mesoporous bimetallic PdPt spheres exhibited superior electrochemical activity compared to commercial Pt black (PtB) and dendritic Pt spheres (with small‐sized pores). Enhanced electrocatalytic performance of trimetallic nanostructure has widely been reported, owing to the electronic effect by introduction of a third element. Therefore, a highly enhanced electrocatalytic activity is expected to introduce to our enlarged mesoporous constructions by simply incorporating a third element. Mesoporous trimetallic Au@PdPt and mesoporous trimetallic PdPtCu spheres with ultra‐large mesopores were synthesized accordingly (**Scheme** **1**). Our mesoporous trimetallic spheres exhibited a higher electrochemical activity and durability for MOR.

**Scheme 1:**
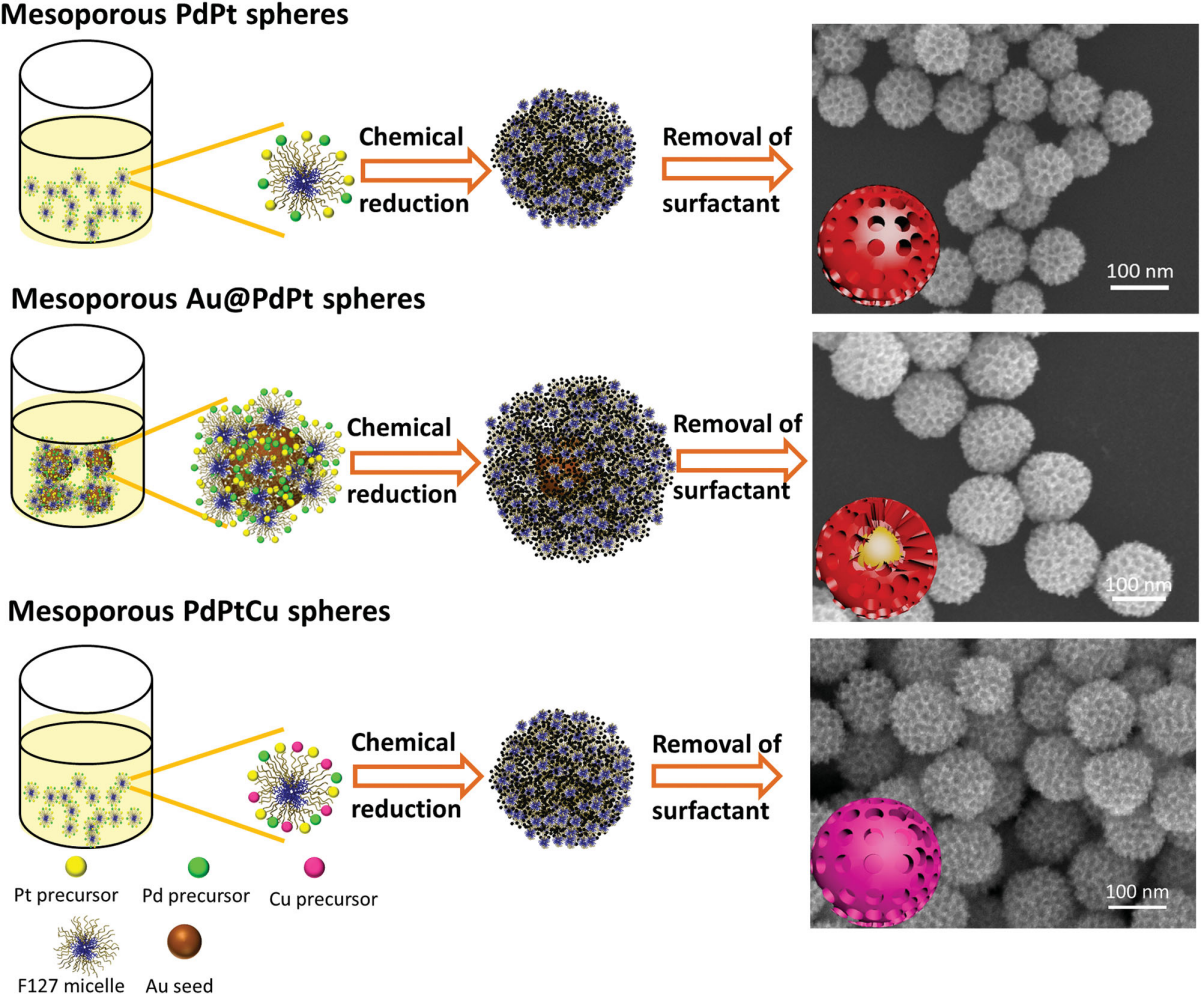
Systematic illustration of mesoporous bimetallic PdPt spheres, mesoporous trimetallic Au@PdPt spheres, and mesoporous trimetallic PdPtCu spheres.

Scanning electron microscope (SEM) was employed to characterize the morphology of as‐prepared sample under the typical condition (**Figure** **1**a). It is clearly revealed that all the particles are fairly uniform in size and shape. Any by‐products, such as nonporous particles, nonspherical shape, or bulks were not observed. Highly magnified SEM image (Figure 1b) shows that well‐defined mesoporous structures are observed over the entire area of the particle surface. The average mesopore size is estimated to be 18.5 ± 0.5 nm with the wall thickness of 8.0 ± 0.5 nm. The mesopores observed on the particle surface are deeply concaved into the center of the spherical particles.

**Figure 1:**
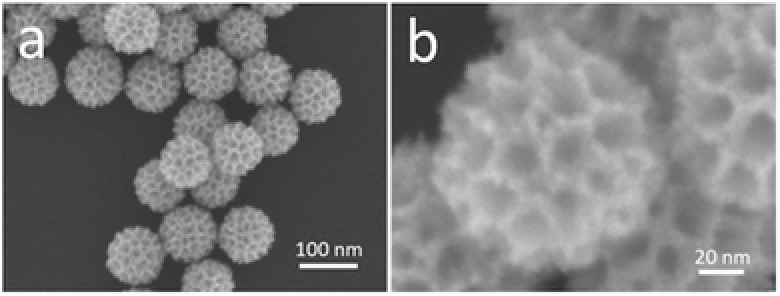
a) Low‐ and b) high‐magnified SEM images of the typical mesoporous bimetallic PdPt spheres obtained at typical conditions.

To confirm the mesostructural periodicity, the typical sample was characterized by low‐angle X‐ray diffraction (XRD) measurement (Figure S1a, Supporting Information). One clear broad peak centered at 2*θ* = 0.33° (*d* = 26.8 nm) supports the formation of periodic mesoporous structure. As seen in SEM images (Figure 1b), the mesopores are closely assembled each other in random manner, although the mesopore size is quite uniform. In this case, the *d*‐spacing (*d* = 26.8 nm) means the pore‐to‐pore distance (i.e., equal to sum of the wall thickness and the mesopore size), which is in agreement with the SEM data (Figure 1b).

For better understanding the distribution of Pd and Pt in the nanoparticles, elemental mapping was investigated for individual sphere (**Figure** **2**). From Figure 2c–e, both Pt and Pd are uniformly distributed. The elemental mapping reveals that most of Pd content is concentrated in the particle center, whereas Pt is placed on the entire sphere. The line‐scanning profiles (Figure 2f) of one sphere more clearly reveal the elemental composition of bimetallic materials, consisting of the higher Pd content at the center, which is in agreement with the elemental mapping results. The wide‐angle XRD peaks (Figure S1b, Supporting Information) are assigned to (111), (200), (220), and (311) diffraction peaks of face‐centered‐cubic (fcc) crystal structure. However, it is very difficult to resolve Pt and Pd crystals in the wide‐angle XRD patterns because of the fact that the lattice mismatch ratio is only 0.77% for Pt/Pd.^11^

**Figure 2:**
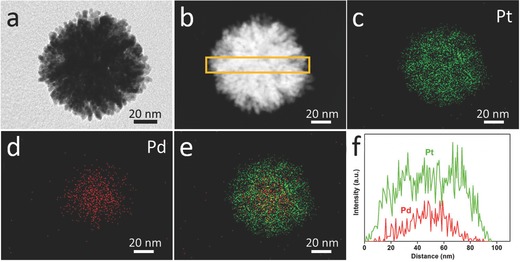
a) TEM image, b) HAADF‐STEM image, c–e) elemental mapping images, and f) compositional line profiles of the mesoporous bimetallic PdPt spheres prepared under ­typical conditions.

Furthermore, the molar ratio of Pt/Pd was confirmed to be 2.66 by the inductively coupled plasma (ICP), which agreed with result obtained by energy‐dispersive X‐ray spectroscope (EDS) (Figure S2, Supporting Information). This value is smaller compared to the starting composition with Pt/Pd of 5.00. All the Pd species dissolved in the precursor solution are reduced to form the cores, while the dissolved Pt species are not completely reduced (i.e., undeposited Pt species are still remained in the solution after the reaction). In the present system, Pd species are more easily and preferentially reduced, although the standard reduction potential for Pt complexes ([PtCl_4_]^2−^/Pt: +0.76 V vs SHE and [PtCl_6_]^2−^/[PtCl_4_]^2−^: +0.68 V vs SHE) is higher than that for ([PdCl_4_]^2−^/Pd: +0.59 V vs SHE). Similar anomalous behavior has been reported for the co‐deposition of Pd and Pt.^12^ Sometimes, the reduction of metal species cannot be interpreted simply according to their standard reduction potentials in the presence of multiple metal precursors and organic additives (e.g., surfactant molecules) due to their complicated reduction kinetics and coordination effect.^13^

As demonstrated above, mesoporous bimetallic PdPt spheres can be synthesized by a simple way in aqueous solution. More control experiments were carried out for understanding the formation mechanism. In general, F127 is well known as one of typical block copolymers and has been utilized as a pore‐directing agent for mesoporous oxide and carbon materials.^14^ Some reports have also demonstrated that F127 can be used as a protecting for preventing the particle aggregations.^15^ As shown in Figure S3a, Supporting Information, nonporous bulk aggregations were formed in absence of F127. When 5 mg of F127 was used, heavily aggregated porous structure was obtained (Figure S3b, Supporting Information), because insufficient F127 cannot inhibit particle aggregation effectively, resulting in formation of irregularly shaped large particles. Further increasing the amount of F127 (60 mg, which is under the typical conditions) resulted in the highly dispersed spheres with mesoporous structure. Thus, it is obvious that the F127 served as the pore‐directing agent as well as protecting agent for preventing the particle aggregations and/or suppressing the random growth of the mesoporous metals.^16^

Interestingly, we found that the particle sizes were easily tuned by varying the molar ratios of H_2_PtCl_6_/K_2_PtCl_4_. **Figure** **3** shows the SEM images and the corresponding particle size distribution histograms of the samples prepared by changing the molar ratios of H_2_PtCl_6_/K_2_PtCl_4_. When K_2_PtCl_4_ was used as the only Pt precursor (i.e., H_2_PtCl_6_/K_2_PtCl_4_ = 0:100), the average particle size was around 46 nm (Figure 3a), which is less than half of size of the sample prepared under the typical condition. When a small amount of H_2_PtCl_6_ was fed together with K_2_PtCl_4_ (i.e., H_2_PtCl_6_/K_2_PtCl_4_ = 10:90), the average ­particle size became 90 nm (Figure 3b). When we increased the molar ratio of H_2_PtCl_6_/K_2_PtCl_4_ from 20:80 to 40:60, the average ­particle size was further increased to 110 nm and well‐defined mesoporous architectures were developed on the particle ­surface (Figure 3c,d). It is noted that further increasing the molar ratio of H_2_PtCl_6_/K_2_PtCl_4_ to 90:10 or 100:0, the reaction cannot take place at 40 °C. Therefore, we increased the ­temperature to 45 °C, resulting in the formation of mesoporous bimetallic PdPt spheres with the average size of 150 and 176 nm, respectively (Figure 3e,f). Even though the molar ratios of H_2_PtCl_6_/K_2_PtCl_4_ were varied, the obtained mesopores sizes were almost constant, which is also an evidence for the formation of surfactant‐directed mesopores.

**Figure 3:**
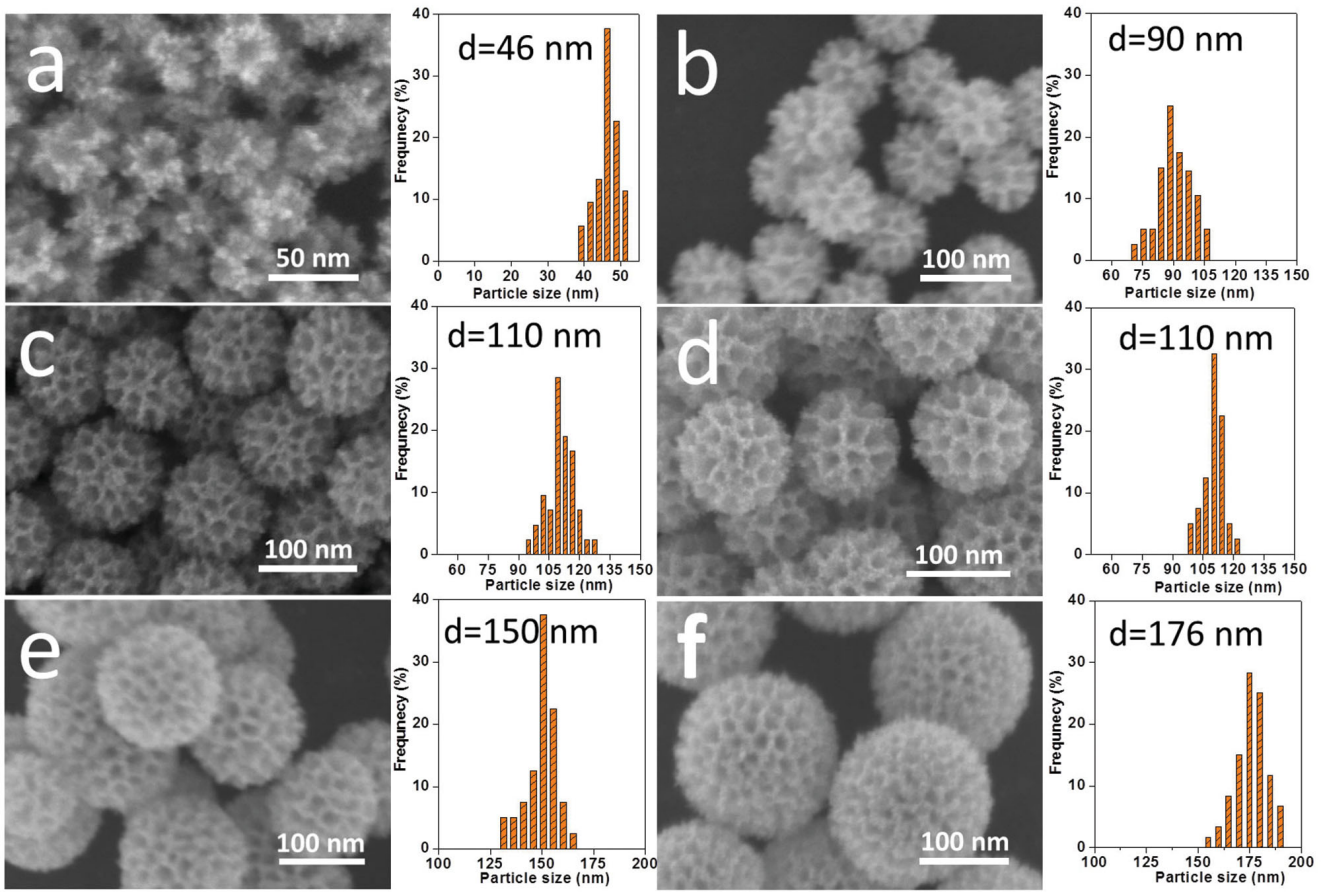
SEM images and particle sizes distributions of the samples prepared by varying the molar ratios of H_2_PtCl_6_/K_2_PtCl_4_: a) 0:100, b) 10:90, c) 20:80, d) 40:60, e) 90:10, f) 100:0.

To further discuss the above particle size control, the reduction rates were visibly monitored (Figure S4, Supporting Information), in which changing the reaction solutions to black means the metal deposition. When only using K_2_PtCl_4_ as precursor, the color of reaction solution was changed from initial yellow to black in 30 min. While using both K_2_PtCl_4_ and H_2_PtCl_6_ as precursors, the solution color gradually changed from yellow to black till 75 min. When only using H_2_PtCl_6_ as precursor, the solution color did not change from yellow to black at 40 °C. From above results, we proposed that the addition of H_2_PtCl_6_ slowed the reduction rate of the metal species. Considering reduction behaviors of two Pt complexes ([PtCl_4_]^2−^/Pt: +0.76 V vs SHE and [PtCl_6_]^2−^/[PtCl_4_]^2−^: +0.68 V vs SHE), it is understandable that [PtCl_6_]^2−^ ions have a lower tendency toward reduction than [PtCl_4_]^2−^ ions. Therefore, by replacing K_2_PtCl_4_ with H_2_PtCl_6_, the deposition rate greatly delays. In general, a balance between nucleation and crystal growth determines the final size of the particles in the products. In the present system, the dissolved Pt and Pd species were reduced by ascorbic acid (AA) at the initial stage. Then, the nuclei were generated and grew further by interaction of the dissolved metal species with AA to form the final products. The slow reduction rate plays a key role in achieving the Pt growth on the Pd nucleus. Therefore, with a decrease in the reduction rate of metal species by addition of H_2_PtCl_6_, the number of nuclei formed at the early stage is thought to be decreased. These nuclei serve as seeds and undergo further crystal growth to afford the final products with larger particle size.

Ascorbic acid is used as a reducing agent, and is oxidized according to the following equilibrium equation: C_6_H_8_O_6_ ↔ C_6_H_6_O_6_ + 2H^+^ + 2*e* ^−^. Thus, in principle the reduction capacity of AA can be decreased by precise control of pH. Hydrochloric acid (HCl) can decrease reduction capacity of AA for retarding reduction of metal precursors, which favors ordered mesoporous spheres. In the present reaction system, we used the optimized amount of HCl to control the crystal growth rate. Figure S5, Supporting Information, shows the morphology of the obtained particles prepared both in the presence and absence of HCl.

Overall, the controls of F127 concentration and compositional ratios of H_2_PtCl_6_ and K_2_PtCl_4_ as Pt precursors, and the use of HCl are responsible for monodispersed mesoporous PdPt spheres with uniform particle sizes. Even when the total amounts of Pt and Pd precursors are two times higher than the typical condition (i.e., the compositional ratios of Pt and Pd precursors are constant.), the pore and the particle sizes are not changed (Figure S6, Supporting Information), indicating the present method has a great potential to large‐scale production for industrial applications.

Many previous studies have proved that high crystallized Pt, especially the Pt surface with kink and step sites, exhibit high activity in the electrocatalysis. Therefore, the crystallinity of mesoporous bimetallic PdPt spheres was studied by high‐resolution transmission electron microscope (TEM). The TEM image of an individual mesoporous bimetallic PdPt sphere reveals the mesoporous structure inside the particle, judging from the significantly different contrast (**Figure** **4**). Unlike mesoporous silica and carbon,^17^ the electron beam cannot pass through the metallic sample. Therefore, it is hard to clearly visualize the mesoporous structure over the entire area. As seen in Figure 4a, the mesopore walls are built by a large quantity of connected nanoparticles. Interestingly, the lattice fringes corresponding to the Pt crystal are coherently extended across over several particles, indicating that the mesopore walls have a good crystallinity. A crystalline grain size of around 7.4 nm was calculated from the (111) diffraction peak based on the Scherrer equation.^18^ Selected‐area electron diffraction (SAED) patterns of an individual sphere (inset of Figure 4a) show several intense spots which are indexed to a fcc crystal structure, suggesting that several grain particles are randomly aggregated in the nanospheres, rather than oriented growth. Some atomic edge and kink sites caused by unsaturated Pt coordination atoms can be observed on the atomic Pt surface, which is highly valuable for high electrocatalytic performance (Figure 4b).^19^

**Figure 4:**
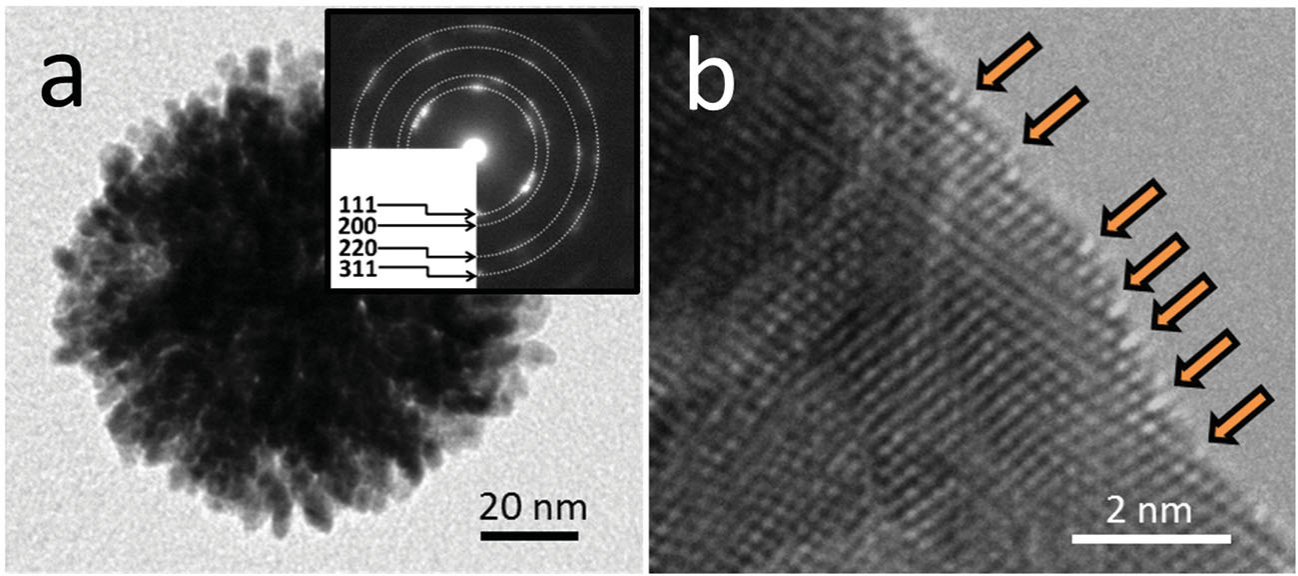
a) TEM and b) HRTEM images of the typical mesoporous bimetallic PdPt spheres. The kink and step sites are indicated by arrows. The SAED patterns obtained from an individual sphere are shown in an inset image in panel (a).

Our method can be extended to synthesize mesoporous trimetallic Au@PdPt spheres (Figure S7, Supporting Information) and mesoporous trimetallic PdPtCu spheres (Figure S8, Supporting Information). The representative SEM, TEM, and HAADF‐STEM images (Figures S7a–c and S8a–c, Supporting Information) reveal that the obtained samples possess well‐defined mesoporous structure and their particle shape and pore size are totally uniform. The average size of spheres is measured to be around 150 nm (for Au@PdPt spheres) and 110 nm (for PdPtCu spheres), respectively. In the case of trimetallic Au@PdPt spheres, we can see a solid core at the center of sphere. The wide‐angle XRD pattern shows two distinct sets of diffraction peaks which can be attributed to fcc crystal structure of Au and Pt/Pd (Figure S7d, Supporting Information). In the case of trimetallic PdPtCu spheres, the XRD pattern shows the metallic fcc structure without other diffraction peaks of monocomponent Pt, Pd, and Cu, indicating that the formation of single‐phase alloy (Figure S8d, Supporting Information). Compared with standard diffraction patterns of Pd and Pt, all the diffraction peaks of PdPtCu slightly shifted to higher angles due to the substitution of the Cu atoms.^20^ From ICP analysis, the atomic ratio of Pd, Pt, and Cu is found to be 37, 38, and 25, respectively. The percentages of Pt and Pd are close to the starting compositional ratio, indicating the Pt and Pd species were completely reduced while the Cu species were not completely reduced. The possible reason is that the Cu^2+^ species have lower reduction potential than those of [PdCl_4_]^2−^ and [PtCl_6_]^2−^.^21^ The compositional ratios of the PdPtCu spheres can be tuned by the amounts of Cu precursor in the synthesis without any noticeable influence to mesoporous structures (Figure S9, Supporting Information).

The electrochemically active surface area (ECSA) of as‐prepared samples can be estimated by measuring the total charge passed during the hydrogen adsorption‐desorption. The specific ECSAs of the mesoporous bimetallic PdPt spheres (Figure 3e), mesoporous trimetallic Au@PdPt spheres (Figure S7a, Supporting Information), mesoporous trimetallic PdPtCu spheres (Figure S8a, Supporting Information), dendritic Pt nanoparticles^22^ (Figure S10, Supporting Information), and PtB are 29.9, 31.6, 26.7, 19.9, and 13.5 m^2^ g^−1^, respectively. To evaluate the electrocatalytic activity of our samples, methanol oxidation reaction (MOR) was selected as a probe reaction. The cyclic voltammetric (CV) measurement was carried out in 0.5 m H_2_SO_4_ containing 0.5 m methanol to investigate the activities of mesoporous bimetallic PdPt spheres, mesoporous trimetallic Au@PdPt spheres and mesoporous trimetallic PdPtCu spheres. The CV curves (Figure S11a,b, Supporting Information) showed the typical features of methanol oxidation on Pt surface. Mesoporous trimetallic Au@PdPt spheres (0.415 A mg^−1^_Pt, 0.311 A mg^−1^_catalyst), trimetallic mesoporous trimetallic PdPtCu spheres (0.430 A mg^−1^_Pt, 0.245 A mg^−1^_catalyst), and mesoporous bimetallic PdPt spheres (0.298 A mg^−1^_Pt, 0.247 A mg^−1^_catalyst) exhibited better catalytic performance than those of dendritic Pt spheres^22^ (with small‐sized and disordered mesopores) (0.152 A mg^−1^_Pt, 0.152 A mg^−1^_catalyst), and commercial PtB (0.087 A mg^−1^_Pt, 0.087 A mg^−1^_catalyst). In general, pure Au, Pd, and Cu themselves are little effective toward MOR in acidic electrolyte. As mentioned above, because of highly miscible of the Pd and Pt atoms, the pseudo Pt–Pd alloy structure should be formed at the internal surface of mesoporous PdPt spheres, which is favorable for reducing the electronic binding energy in Pt and improving catalytic activity.^23^ Also, it is clear that the introduction of the third element (Au or Cu) indeed plays an important role for further enhancing the electrocatalytic activity toward MOR because a moderate decrease of CO adsorption is caused by incorporating Au or Cu into the catalysts.[[qv: 13b]],^24^ As a result, both of our mesoporous bimetallic PdPt spheres, mesoporous trimetallic Au@PdPt, and mesoporous trimetallic PdPtCu spheres exhibited a higher activity compared to dendritic Pt and PtB catalysts. Even when the activity was normalized by ECSAs, our mesoporous, Au@PdPt (1.31 mA cm^−2^_Pt), PdPtCu (1.61 mA cm^−2^_Pt), and PdPt (0.997 mA cm^−2^_Pt) samples still exhibited higher activity than that of dendritic Pt (0.764 mA cm^−2^_Pt) nanoparticles and PtB (0.643 mA cm^−2^_Pt). A probable reason for the superior activity is a large amount of kink and step sites created on the mesoporous surfaces (Figure 4b). This benefits to breaking CH bonds in methanol decomposition, thereby an improved catalytic performance was observed in MOR.^25^

Another important reason for improving the electrochemical activity is that the ultra‐large mesoporous structure can accelerate the transport for reactants and products. As shown in Figure S12c, Supporting Information, in proportion to the scan rate, the forward oxidation peak current densities of mesoporous PdPt spheres and dendritic Pt spheres are increased linearly with the square root of the scan rate, suggesting that the oxidation of methanol at electrodes is a diffusion‐controlled process. The slope of mesoporous PdPt spheres is significantly larger than that of dendritic Pt spheres, revealing that the diffusion efficiency of methanol molecules inside large‐sized pores is higher than that inside small‐sized and dendritic pores.^26^ As an effective catalyst, the stability is a very important factor for MOR. Thus, the stability of catalysts were also investigated by chronoamperometric measurement at 0.6 V for 3000 s in 0.5 m H_2_SO_4_ containing 0.5 m CH_3_OH. It is obvious that our mesoporous catalysts possess excellent stability during the electrochemical measurement, in comparison to dendritic Pt spheres and commercial PtB catalyst (Figure S11c,d, Supporting Information) in a long period of 3000 s.

In summary, we have successfully developed a facile synthetic method for preparation of multimetallic mesoporous spheres with ultra‐large mesopores. In the synthesis, the concentration of F127, and reduction kinetics of metal species are critical to formation of uniform ultra‐large mesoporous structure. Because of the high surface area, large‐sized mesopores, our mesoporous spheres exhibit enhanced electrochemical activity for MOR. This present approach, based on a green and environment‐friendly process in dilute surfactant solution, could be extended to the synthesis of other metal and alloy system with ultra‐large mesopores for high‐performance ­catalysts and also will be used as a promising approach for large‐scale industrial production.

## Supporting information

As a service to our authors and readers, this journal provides supporting information supplied by the authors. Such materials are peer reviewed and may be re‐organized for online delivery, but are not copy‐edited or typeset. Technical support issues arising from supporting information (other than missing files) should be addressed to the authors.

Supplementary
