# Supplementary material for: Multimetallic Mesoporous Spheres Through Surfactant‐Directed Synthesis
Source: Adv Sci (Weinh). 2015 Jun 25;2(8):1500112. doi: 10.1002/advs.201500112 (PMC5115414; doi:10.1002/advs.201500112)
Supplement: Supplementary file 1 — Supplementary [file ADVS-2-0j-s001.pdf]

Copyright WILEY-VCH Verlag GmbH & Co. KGaA, 69469 Weinheim, Germany, 2015.

ADVANCED SCIENCE
Open Access

Supporting Information

for *Adv. Sci.*, DOI: 10.1002/advs. 201500112

Multimetallic Mesoporous Spheres Through Surfactant-Directed Synthesis

Bo Jiang, Cuiling Li, Masataka Imura, Jing Tang, and Yusuke Yamauchi**

Experimental section.

Materials. All chemicals and solvents were used as received without further purification. H_2PtCl_6, K_2PtCl_4, Na_2PdCl_4, $NaBH_4$, ascorbic acid (AA), cetyltrimethylammonium bromide (CTAB), and methanol were purchased from Nacalai Tesque, Inc. (Kyoto, Japan). Pluronic F127 and nonionic surfactant Brij58 were obtained from Sigma-Aldrich. Commercial PB (HiSPEC®1000) was supplied by Johnson Matthey Company. All solutions were prepared with deionized water treated with a Millipore water purification system (Millipore Corp.).

Preparation of mesoporous bimetallic PdPt spheres. In a typical synthesis of mesoporous bimetallic PdPt spheres, 0.6 ml of Na_2PdCl_4 solution (20.0 mM), 1.2 ml of K_2PtCl_4 solution (20.0 mM), 1.8 ml of H_2PtCl_6 solution (20.0 mM), 60.0 μl of HCl solution (6.0 M), and 60.0 mg of Pluronic F127 were mixed. After F127 was completely dissolved, 3.0 ml of ascorbic acid (AA) solution (0.1 M) was added to the above solution, giving the final Na_2PdCl_4, K_2PtCl_4, H_2PtCl_6 precursor amounts of 0.012, 0.024, and 0.036 mmol, respectively. In the typical condition, the relative mole ratio of Pt/Pd species was 5.00. Then, the mixed solution was continuously sonicated in a water bath for 4 hours at 40 ^{o}C. Finally, the sample was collected by centrifugation at 14,000 rpm for 20 min and the residual Pluronic F127 was removed by three consecutive washing/centrifugation cycles with ethanol and water.

Preparation of mesoporous trimetallic Au@PdPt spheres. The procedure of synthesis of mesoporous trimetallic Au@PdPt spheres was similar as synthesis of mesoporous bimetallic PdPt spheres except adding the Au nanoparticles. Firstly, Au nanoparticles with 45 nm in diameter were synthesized. An aqueous solution (10.0 ml) containing $HAuCl_4$ (0.25 mM) and CTAB (75.0 mM) was prepared in a flask. To this mixture, 0.6 ml of ice-cold $NaBH_4$ solution (10.0 mM) was rapidly injected with stirring for 3 hours. Then, the obtained suspension was diluted 100 times with water. The obtained seed solution (0.3 ml) was mixed with 25.0 ml of the reaction solution containing 2.0 ml of CTAB solution (0.2 M), 0.1 ml of $HAuCl_4$ solution (40.0 mM), and 2.0 ml of AA solution (0.1 M), and water. After that, the above solution was kept in room temperature for 2 hours. Finally, CTAB-coated Au nanoparticles were separated from excess CTAB by washing with water followed by centrifugation twice at 9000 rpm.

For coating mesoporous PdPt shell, 0.6 ml of Na_2PdCl_4 solution (20.0 mM), 1.2 ml of K_2PtCl_4 solution (20.0 mM), 1.8 ml of H_2PtCl_6 solution (20.0 mM), 60.0 μl HCl solution (6.0 M), and 60.0 mg of Pluronic F127 were mixed together. After F127 was completely dissolved, the as-prepared Au seeds (0.01 mmol) and 3.0 ml ascorbic acid solution (0.1 M) was added to the above solution. Then, the mixed solution was continuously sonicated in a water bath for 4 hours at 40 ^{o}C. Finally, the sample was collected by centrifugation at 14000 rpm for 20 min and the residual Pluronic F127 was removed by three consecutive washing/centrifugation cycles with ethanol and water.

Preparation of mesoporous trimetallic PdPtCu spheres. Firstly, 1.5 ml Na_2PdCl_4 (20.0 mM), 1.5 ml H_2PtCl_6 (20.0 mM), 1.5 ml $CuCl_2$ (20 mM), 0.2 ml HCl (6.0 M) and 50.0 mg Pluronic F127 were mixed. After F127 was dissolved under ultrasound condition, an aqueous solution of 2.0 ml ascorbic acid (0.1 M) was added to above solution, giving the final Na_2PdCl_4, H_2PtCl_6, $CuCl_2$ precursor amounts of 0.03, 0.03, and 0.03 mmol, respectively. Then, the mixed solution was kept in an oil bath for 4 hours at 95 ^{o}C. Finally, the sample was collected by centrifugation at 14000 rpm for 20 min and the residual Pluronic F127 was removed by several consecutive washing/centrifugation cycles with ethanol and water. The as-prepared sample was stored in ethanol until the time of use and then dried at room temperature.

Preparation of dendritic Pt nanoparticles. Firstly, an aqueous solution consisting of nonionic Brij58 surfactant was prepared under stirring. Then, K_2PtCl_4 and ascorbic acid (AA) were added to the surfactant Brij58 solutions, making the final concentration of K_2PtCl_4 and AA to be 5 mM and 12.5 mM, respectively. The final concentration of Brij58 in solution was 0.5 %. Then, the reaction solution was incubated without any external treatment for 12 h at room temperature. Finally, the sample was collected by centrifugation at 14000 rpm for 20 min and the residual Brij58 was removed by several consecutive washing/centrifugation cycles with ethanol and water. The as-prepared sample was stored in ethanol and then dried at room temperature.

Materials characterization. Scanning electron microscope (SEM) observation was carried out using a Hitachi SU-8000 microscope operated at 5.00 kV. Transmission electron microscope (TEM) and high-angle annular dark-field scanning TEM (HAADF-STEM) observations were carried out using a JEOL JEM-2100F operated at 200 kV equipped with an energy-dispersive spectrometer. The samples for TEM study were prepared by depositing a drop of the diluted colloidal suspension on a carbon-coated copper grid. A wide-angle powder X-ray diffraction (XRD) pattern was recorded with a Rigaku Rint 2500 diffractometer with monochromated Cu Kα radiation. Low-angle XRD patterns were recorded by using a NANO VIEWER (Rigaku, Japan). Both CV and chronoamperometric experiments were performed using a CHI 842B electrochemical analyzer (CHI Instruments, USA). A conventional three-electrode cell was used, including an Ag/AgCl (containing saturated KCl) electrode as a reference electrode, a platinum wire as a counter electrode, and a modified glassy carbon electrode (GCE) as a working electrode. The modified GCE was coated with as-produced samples (5.0 μg) and dried at room temperature. Then, 5.0 μL of Nafion (0.05 wt %) was coated on the surface of the modified GCE and dried before electrochemical experiments. Prior to electrochemical experiments, the GCEs modified with the samples were activated electrochemically by cycling the electrode potential between -0.2 V and +1.5 V (*vs.* Ag/AgCl) in 0.5 M H_2SO_4 until CVs that were characteristic for a clean Pt electrode were obtained.

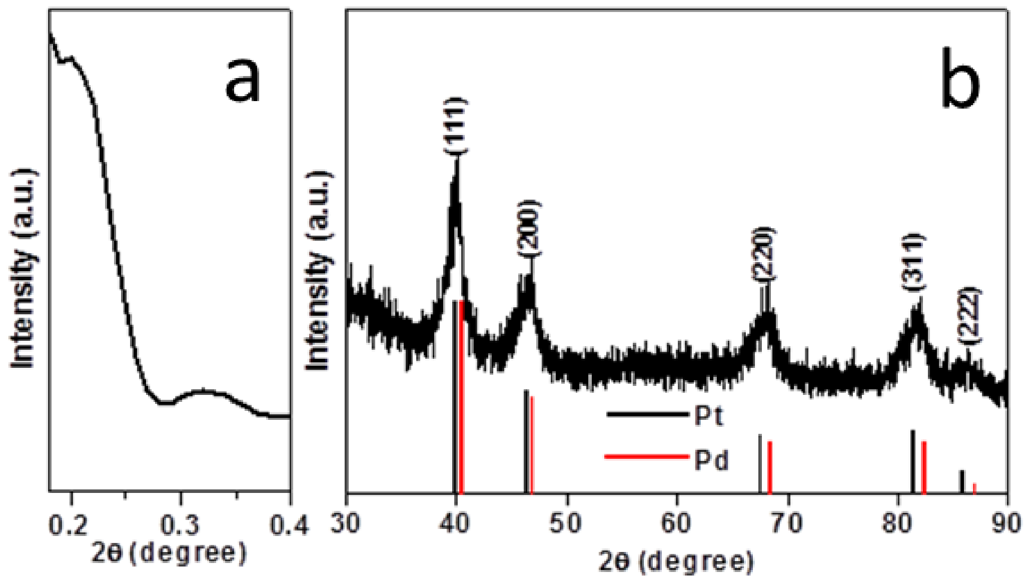

Fig. S1. (a) Low-angle and (b) wide-angle XRD patterns of the mesoporous bimetallic PdPt spheres prepared under typical conditions.

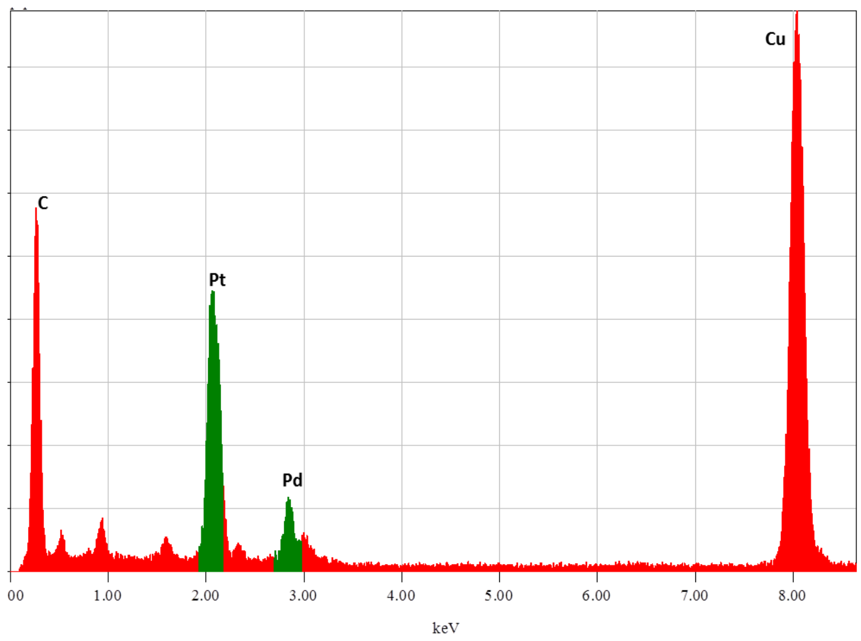

Fig. S2. Electron dispersive X-ray spectroscopy of the mesoporous bimetallic PdPt spheres.

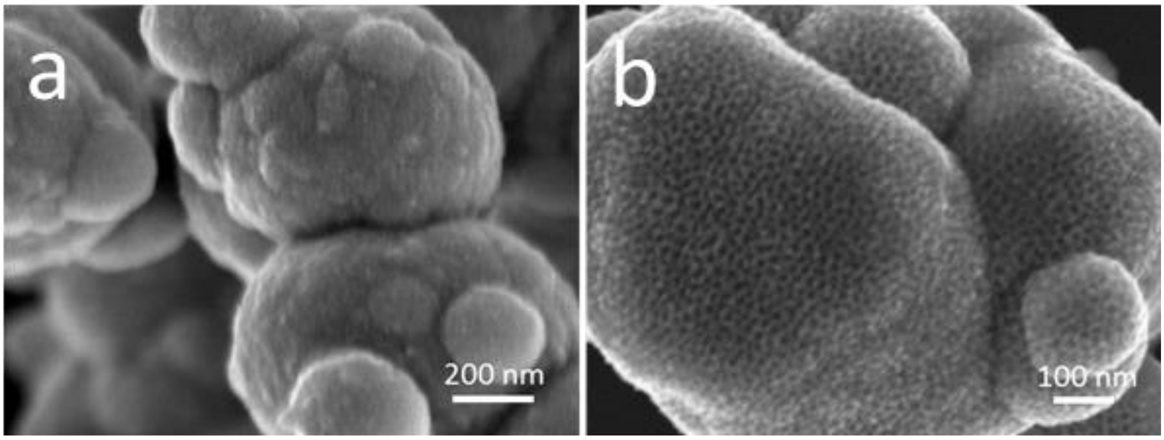

Fig. S3. SEM images of samples prepared (a) without F127 and (b) with 5.0 mg F127.

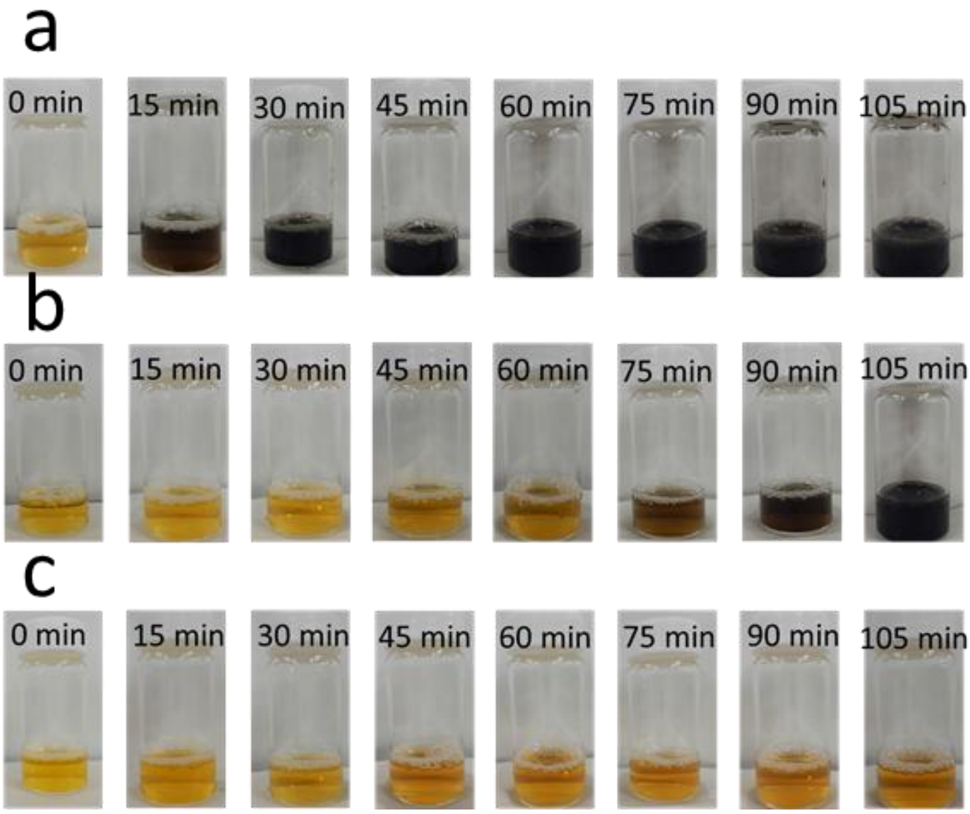

Fig. S4. Pohotgraphs of colloidal suspensions taken at different reaction times at 40 ^{o}C ((a) H_2PtCl_6/K_2PtCl_4=0:100, (b) H_2PtCl_6/K_2PtCl_4=60:40 and (c) H_2PtCl_6/K_2PtCl_4=100:0, respectively).

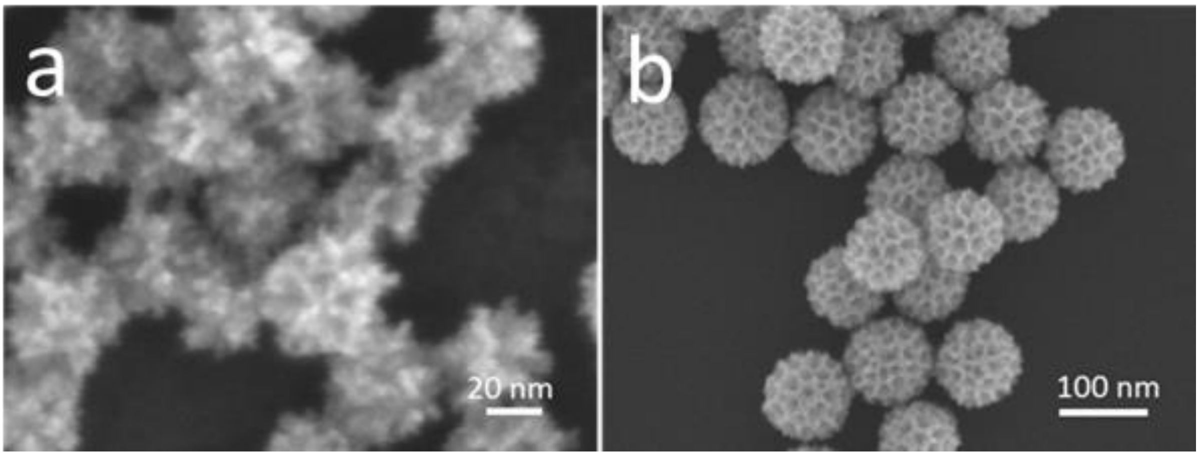

Fig. S5. SEM images of samples prepared (a) without HCl and (b) with HCl.

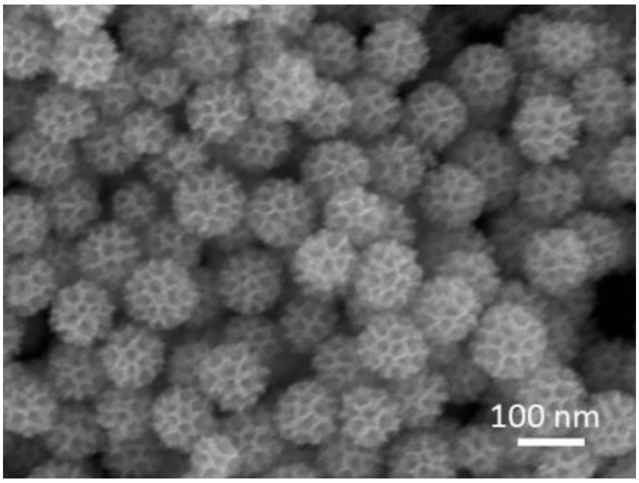

Fig. S6. SEM image of sample prepared by increasing the amount of platinum and palladium precursors.

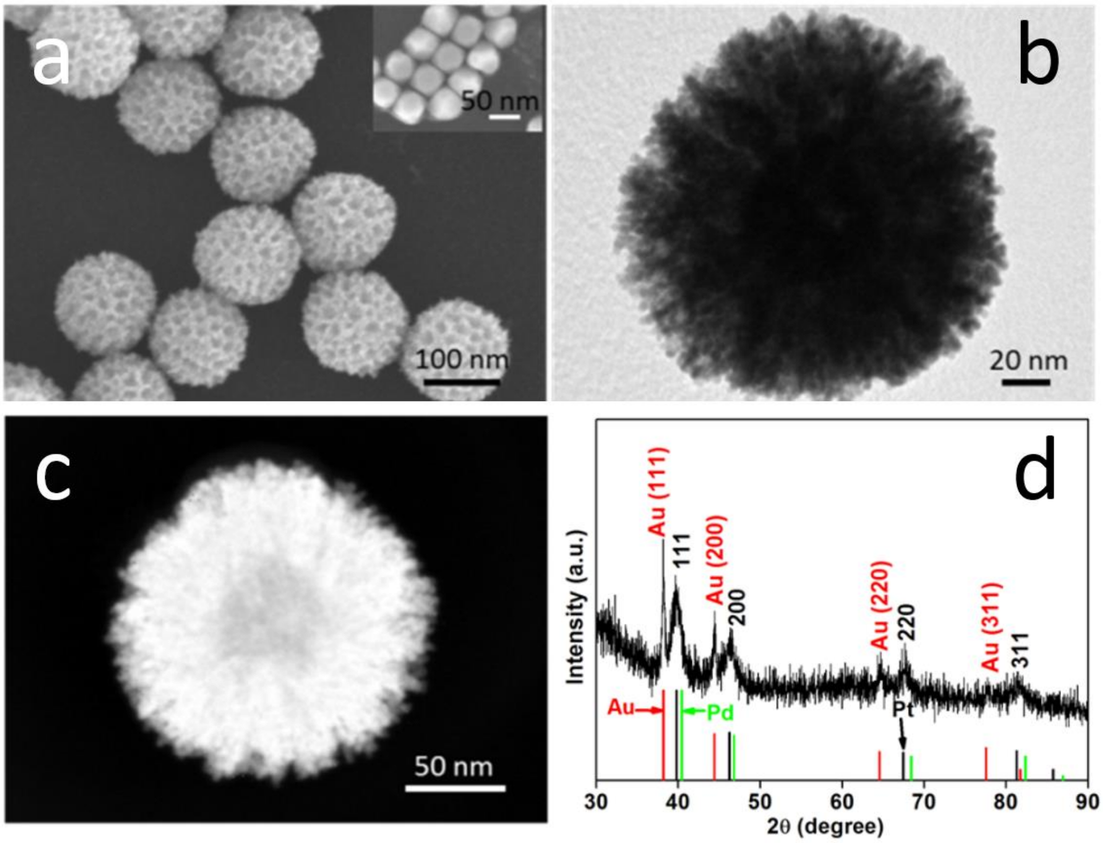

Fig. S7 (a) SEM, (b) TEM, (c) HAADF-STEM images, and (d) wide-angle XRD pattern of mesoporous trimetallic Au@PdPt spheres. Inset image in panel (a) shows the as-prepared Au seeds.

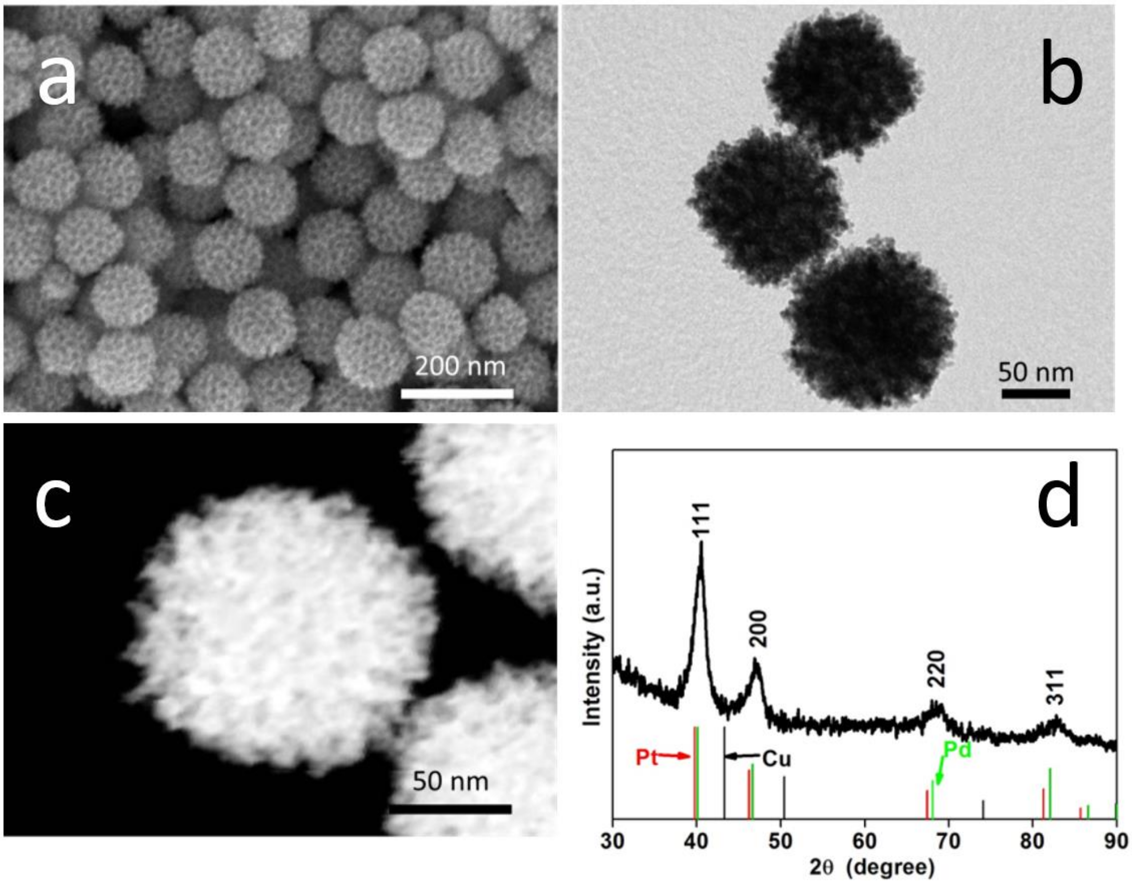

Fig. S8. (a) SEM, (b) TEM, (c) HAADF-SETM images, and (d) wide-angle XRD patterns of mesoporous trimetallic PdPtCu spheres.

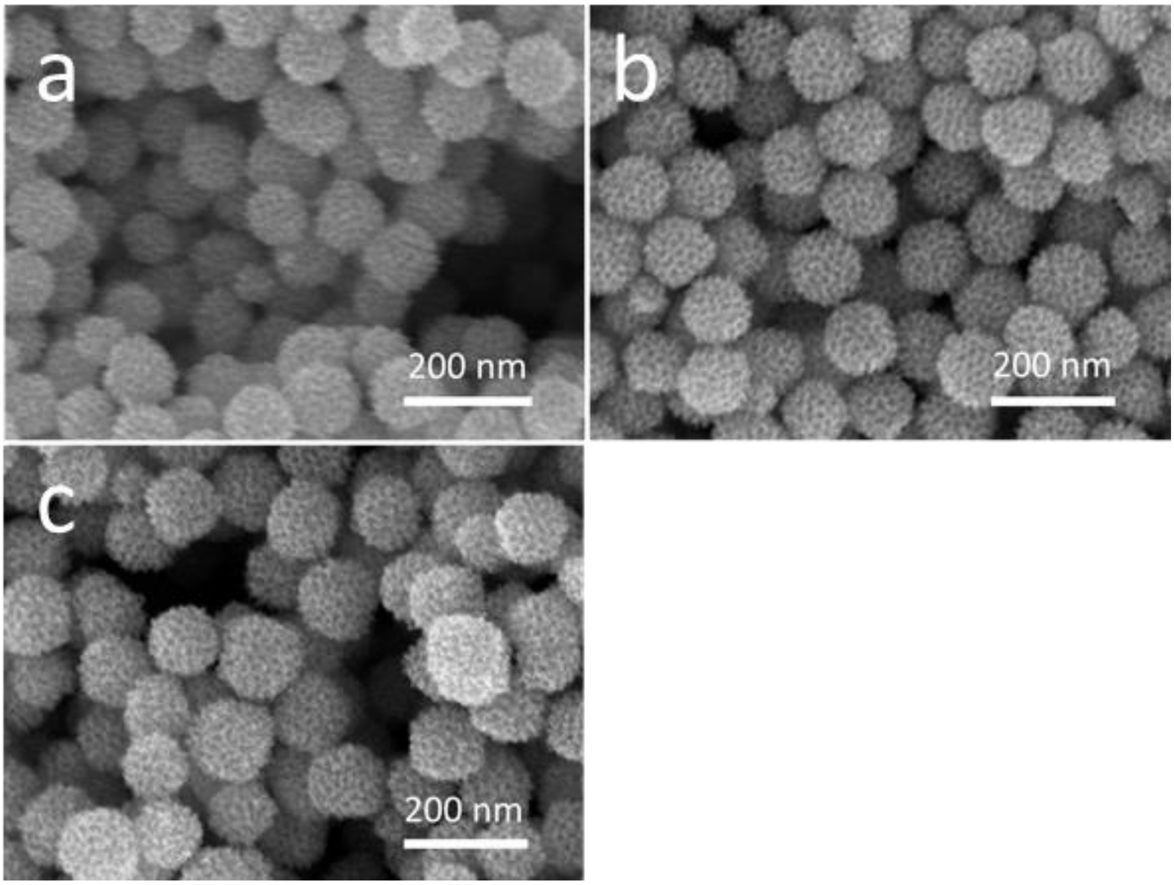

Fig. S9. SEM images of the mesoporous trimetallic PdPtCu spheres with different amount of Cu. (a) mesoporous trimetallic $Pd_{44}Pt_{44}Cu_{12}$, (b) mesoporous trimetallic $Pd_{37}Pt_{38}Cu_{25}$, (c) mesoporous trimetallic $Pd_{27}Pt_{30}Cu_{43}$.

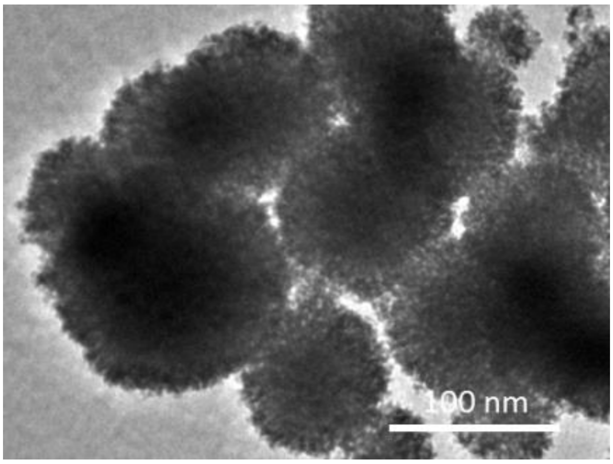

Fig. S10. TEM image of the dendritic Pt particles prepared.

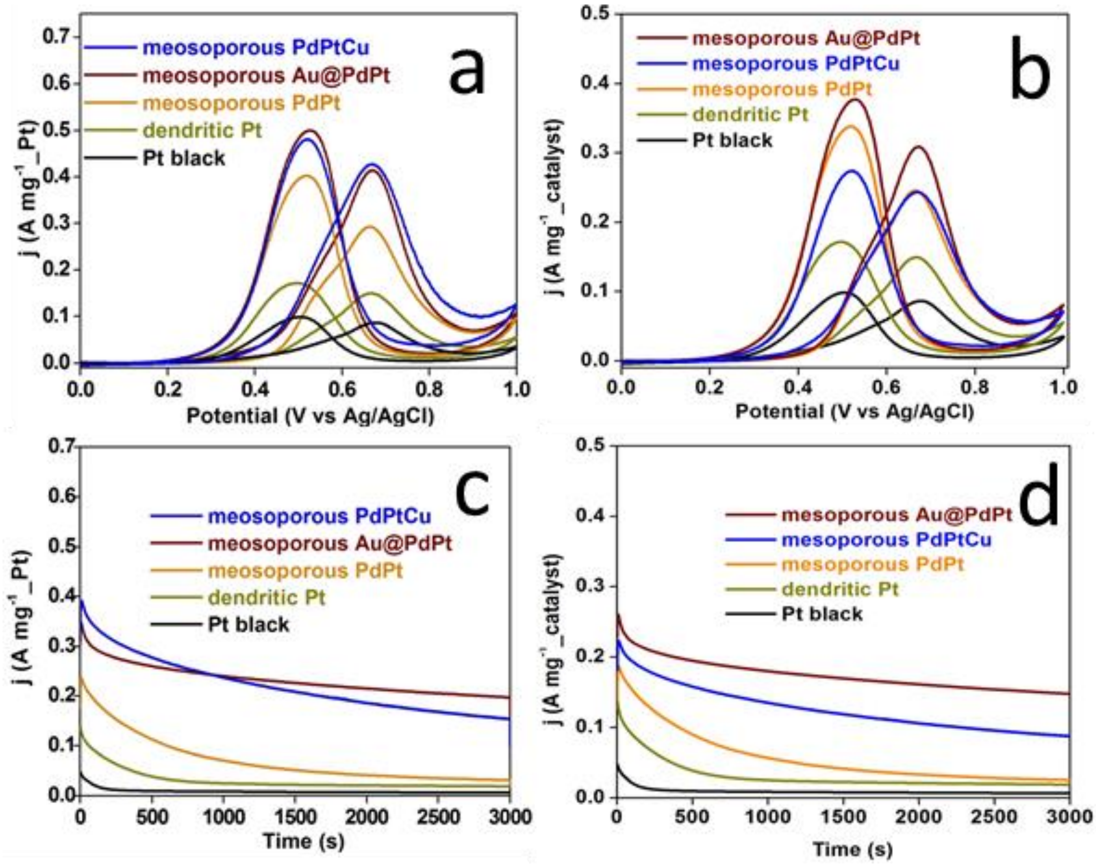

Fig. S11. (a, b) Cyclic voltammograms and (c, d) Chronoamperometric curves (recorded at 0.6 V) for MOR catalyzed by different catalysts in 0.5 M H_2SO_4 containing 0.5 M methanol. The current densities are normalized by (a, c) the Pt mass and (b, d) the total catalyst mass.

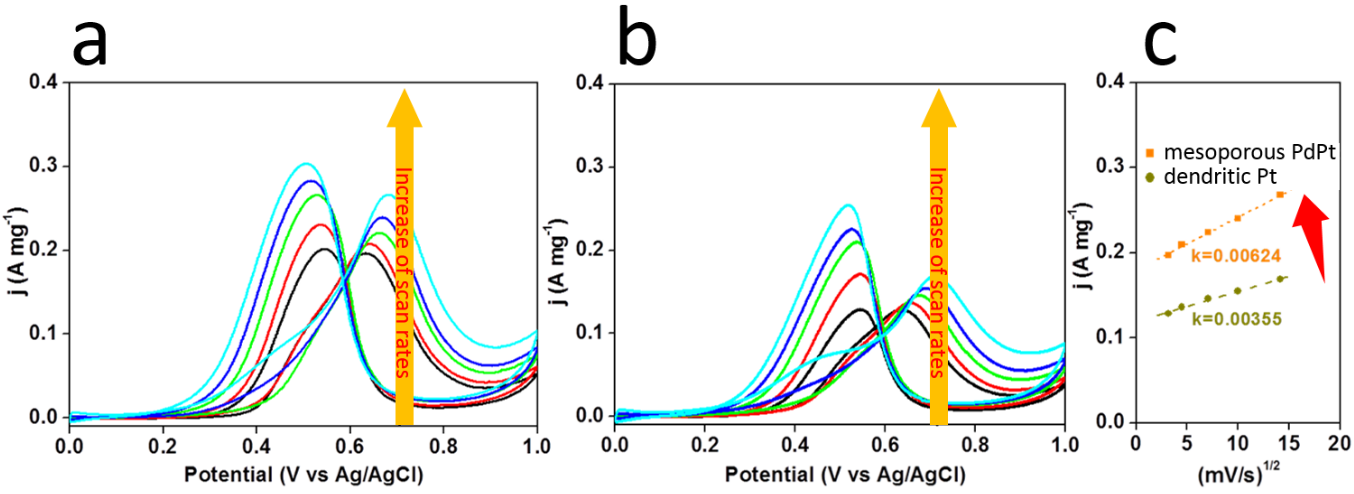

Fig. S12. Cyclic voltammograms of (a) mesoporous PdPt spheres (with large-sized pores) and (b) dendritic Pt catalyst (with small-sized pores) at scan rate of 10, 20, 50, 100, 200 mV/s. (c) the relationship of forward oxidation peak current density vs. scan rate for the mesoporous PdPt sphere and dendritic Pt catalyst.
